# Case report: synovial sarcoma of the axilla with brachial plexus involvement

**DOI:** 10.1186/s12957-018-1466-7

**Published:** 2018-08-13

**Authors:** Ziyad M. Mohaidat, Abed-Allah A. Saleh, Salah Al-gharaibeh, Ibrahim R. Yousef

**Affiliations:** 1 Orthopedic Surgery Division, Special Surgery Department, Faculty of Medicine, Jordan University of Science and Technology, Irbid, 22110 Jordan; 2 Orthopedic Department, King Abdullah University Hospital, Irbid, Jordan; 3 Jordan University of Science and Technology and King Abdullah University Hospital, Irbid, Jordan

**Keywords:** Axilla, Synovial sarcoma, Brachial plexus, Deltopectoral approach

## Abstract

**Background:**

Synovial sarcoma is a rare soft tissue sarcoma which most commonly affects the extremities of young adults. Axilla involvement by this sarcoma is very rare especially with involvement of the brachial plexus. This combination adds to the challenge in approaching such tumors which might significantly affect survival and function.

**Case presentation:**

Herein, we present a 48-year-old female patient who presented with an isolated painless lump in her right axilla. Initially, her workup, looking for possible breast cancer, included fine-needle aspiration (FNA) which did not provide the diagnosis. Core-needle biopsy, performed later, revealed monophasic synovial sarcoma. Her workup studies revealed no metastasis. Then, through extensile deltopectoral approach, the tumor was dissected out from within the brachial plexus. Ulnar nerve was sacrificed in order not to compromise the surgical margins which were confirmed tumor free by final pathology. The patient did not receive chemotherapy or radiation upon consultations with medical and radiation oncology teams. Her follow-up revealed no tumor recurrence with no restriction of her right shoulder motion.

**Conclusion:**

Our case report represents a very rare occurrence of synovial sarcoma in the axilla with involvement of the brachial plexus. When clinical and radiological findings are suggestive of soft tissue sarcoma of the axilla, we recommend getting core-needle biopsy rather than fine-needle aspiration for earlier diagnosis. Early referral and multidisciplinary approach may contribute to better management.

## Background

Synovial sarcoma is rare, representing about 5–8% of all soft tissue sarcomas [1, 2]. It usually affects the extremities of young patients around the age of 35 years [1, 3]. Although called synovial sarcoma, it is rarely intra-articular [4].

Synovial sarcoma diagnosis is based on immuno-histochemical examination since it most commonly presents as a lump with no clinical or radiological diagnostic features [2]. Translocation (X; 18) is characteristic in about 90% of cases [3, 4].

The treatment of synovial sarcoma is usually a combination of wide surgical excision followed by adjuvant radiotherapy [1, 3, 4]. The role of chemotherapy is controversial as either a neoadjuvant or an adjuvant therapy [1, 3, 4].

The case presented here represents a very rare occurrence of synovial sarcoma of the axilla with involvement of the brachial plexus adding to the challenging anatomical considerations of both the axilla and brachial plexus. We are describing the different clinical and radiological features of the patient together with the management approach.

## Case presentation

This is a 48-year-old female patient who was referred after a biopsy of her right axillary mass revealed synovial sarcoma.

The patient incidentally noticed a painless mobile lump in her right axilla about 6 months prior to her biopsy. The mass did not change in size over this period. It was associated with increasing numbness involving her right ring and little fingers that was bothering her during her daily activities especially during the last 2 months. She reported no other masses in her breasts or elsewhere.

Her referring surgeon evaluated her initially for the possibility of breast cancer by ultrasound and mammogram; both of which were negative. So, he performed FNA initially which was inadequate. Then, core-needle biopsy was performed which revealed monophasic synovial sarcoma.

Physical examination of the right axilla showed: 5 × 5 cm ill-defined mobile mass with smooth surface located in the apex of the axilla with no overlying skin changes. No adjacent masses or regional lymph nodes including axillary and cervical lymph nodes were felt. Her peripheral neurovascular examination was unremarkable except for mild decrease in superficial touch sensation in her right little and ring fingers.

Staging studies were performed including MRI of the axilla and CT angiogram for local vascular assessment. CT of the chest, abdomen, and pelvis with bone scan showed no evidence of metastasis.

**Fig. 1:**
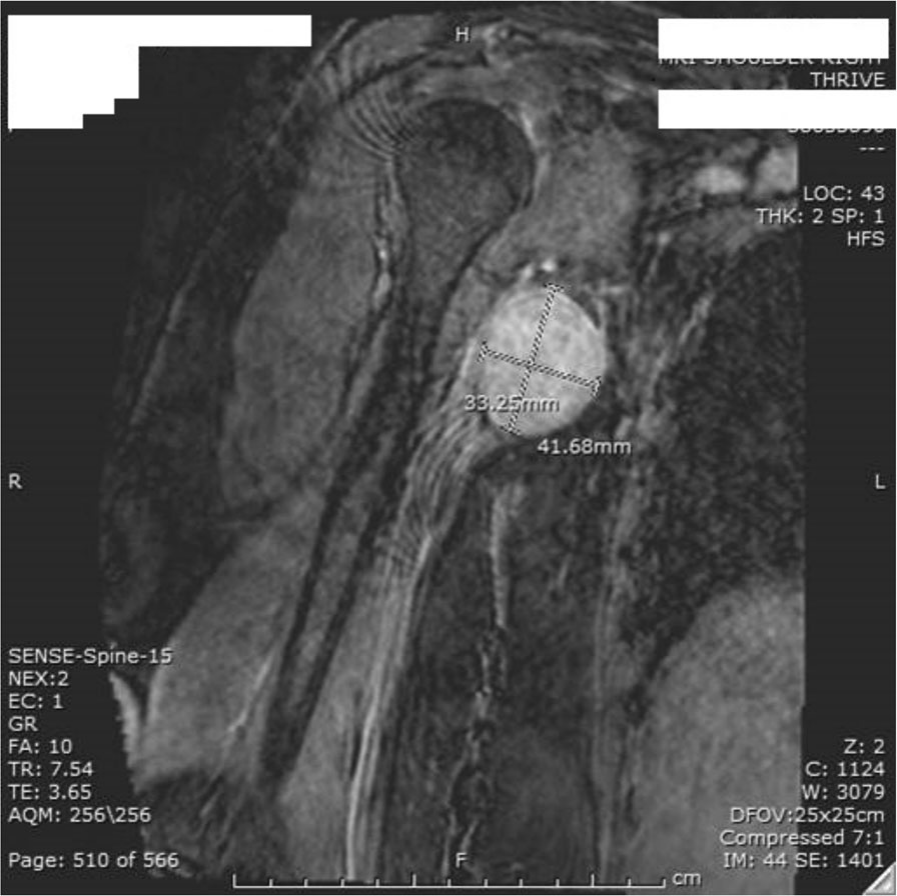
MRI (coronal view of the heterogeneous axillary mass)

**Fig. 2:**
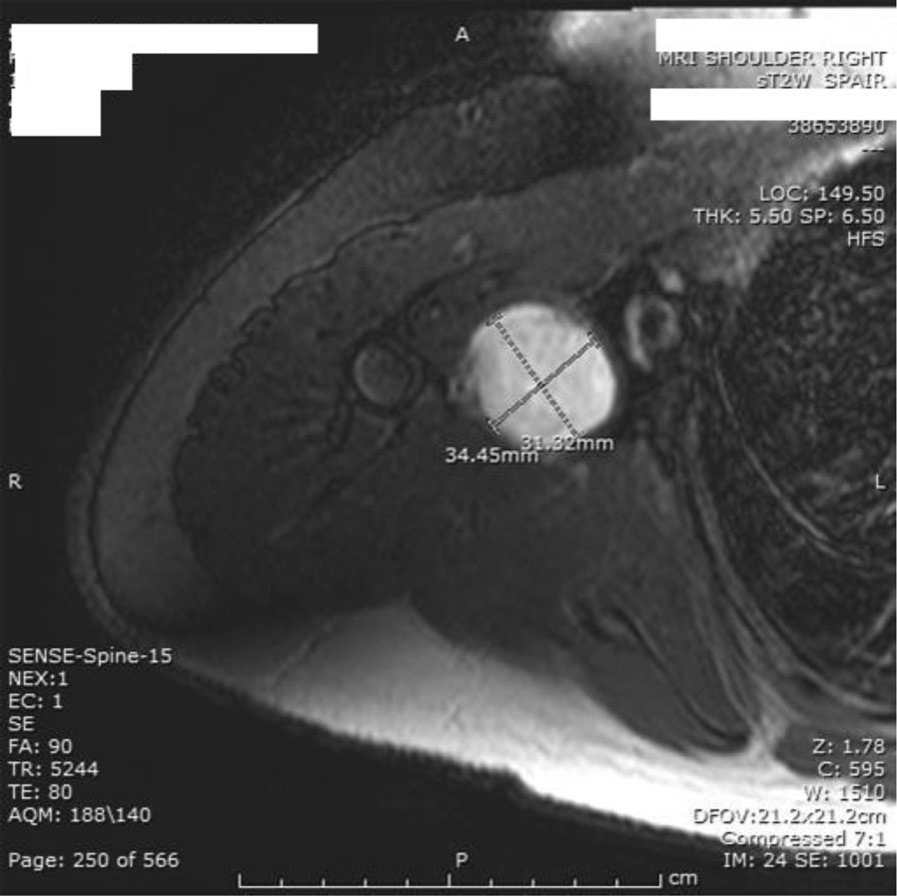
MRI (axial view) showing 3 × 3 cm mass displacing the axillary artery

MRI showed well-defined oval-shaped heterogeneous soft tissue mass in close proximity to the axillary artery with no definite encasement. The mass measured 3 × 3.3 cm in axial diameter and 4 × 4 cm craniocaudally (Figs. 1 and 2). The mass was isointense on T1 and slightly hyper intense on T2 with vivid enhancement post-gadolinium administration.

**Fig. 3:**
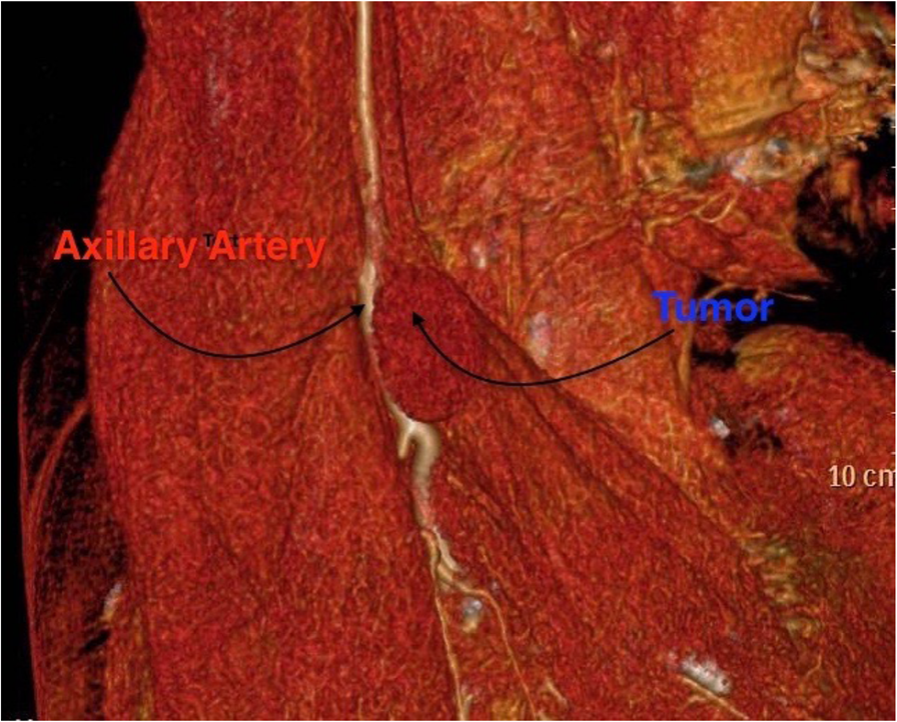
CT angiogram showing the tumor compressing the axillary artery

CT angiogram showed mild mass compression at axillary/brachial arteries transition with patent peripheral vessels (Fig. 3).

Based on the radiological and the histopathological findings, the plan was to proceed with mass excision after exploration of the axillary artery and the surrounding brachial plexus. However, forequarter amputation and the need for adjuvant radiation were to be considered subsequent to the surgery.

The patient provided consent for the plan, and she was made aware that some neurological deficit is to be expected after surgery among other complications.

**Fig. 4:**
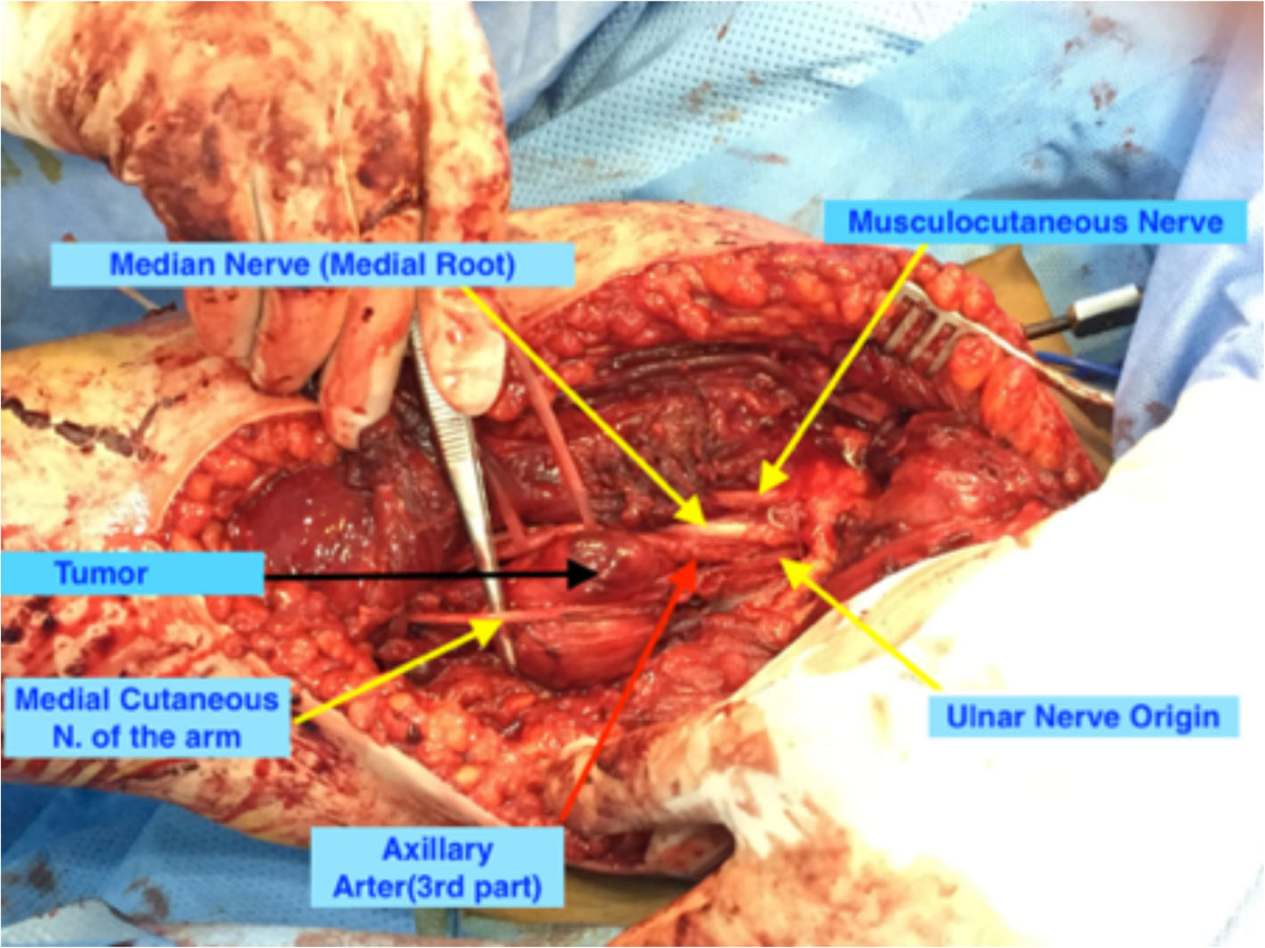
Proximal extension of the tumor in between the axillary artery, the medial root of the median nerve and the origin of the ulnar nerve

**Fig. 5:**
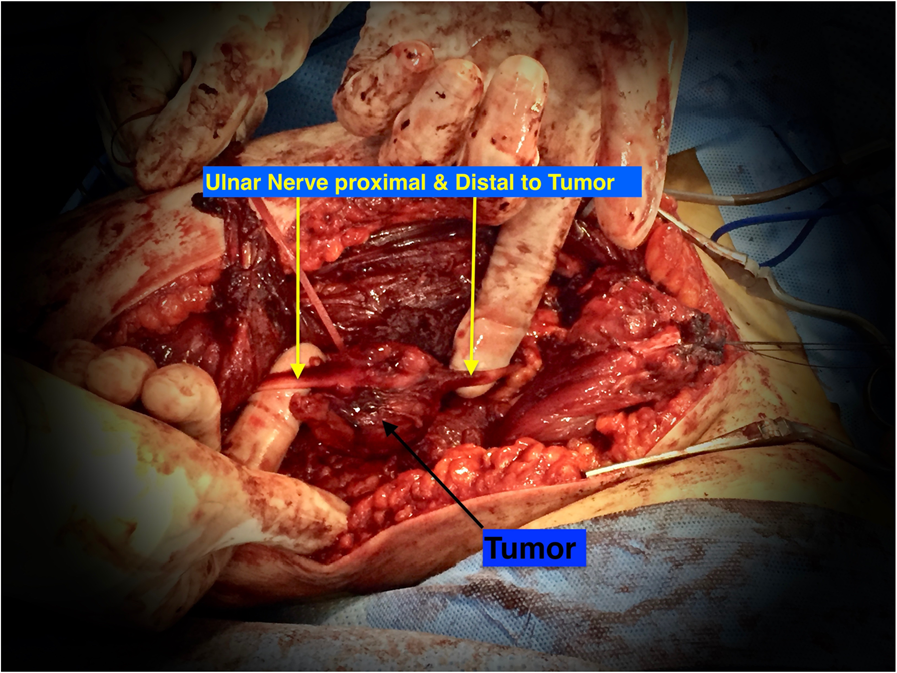
The ulnar nerve was intimately adherent to the tumor

The tumor was exposed through extensile deltopectoral approach after developing intermuscular planes and reflecting pectoralis muscles. The tumor was located anteromedial to the third part of the axillary artery pushing the medial root of the median nerve anterolaterally and the origin of the ulnar nerve medially (Fig. 4). We were able to dissect out the axillary artery and the median nerve easily away from the tumor. However, the ulnar nerve was intimately adherent to the tumor capsule, so we decided to sacrifice it to achieve tumor free margin (Fig. 5). Grossly, the tumor capsule was dissected out completely. No other masses were detected in the surgical bed.

The patient had unremarkable postoperative course. However, she lost her ulnar nerve function with loss of sensation involving her right little and ring fingers.

Her final histopathology revealed monophasic synovial sarcoma with microscopically free margins. The patient did not receive postoperative radiation. Upon follow-up, 9 months after surgery, no clinical or radiological recurrence was observed.

## Discussion

Our patient represents a unique case of axillary synovial sarcoma arising from within the brachial plexus. This combination adds to the challenge in approaching such tumors which might significantly affect survival and function. This localization represents a very rare occurrence. In a study of a large referral population with malignant soft tissue tumors by Kransdorf [2], only 3% (32 out of 1093 patients) of axillary and shoulder malignant soft tissue tumors were synovial sarcoma.

Synovial sarcoma involving the brachial plexus is extremely rare. Upon review of multiple series of brachial plexus tumors [5–9] with a total of 524 patients, only two patients with the diagnosis of synovial sarcoma were detected [6, 9].

The patient’s age is older than the usual age of occurrence between 15 and 40 years [4]. Older age can indicate a worse prognosis [3].

There might have been a delay in the diagnosis and treatment of this patient. This delay might be related to the rarity of synovial sarcoma especially in the axillary region; hence, it was not included in the differential diagnosis at least initially. The initial fine-needle aspiration (FNA) added to the delay as it was inconclusive for the diagnosis. Core-needle biopsy could have provided earlier diagnosis since it might be more helpful for the diagnosis of soft tissue sarcoma [10].

The possible role of neoadjuvant chemotherapy was discussed with the medical oncology team. Recognizing the size and location of the lesion, together with the controversial role of chemotherapy in the treatment of adult synovial sarcoma [1, 3, 4] led to the decision that neoadjuvant chemotherapy, most likely, will not be of benefit in this situation.

Our patient did not receive postoperative radiation. The decision was made based on the size of the tumor being less than 5 cm and on the postoperative histopathological findings of tumor-free resection margins.

## Conclusions

In conclusion, our case report represents a very rare occurrence of synovial sarcoma in the axilla with involvement of the brachial plexus. When clinical and radiological findings are suggestive of soft tissue sarcoma of the axilla, we recommend getting core-needle biopsy rather than fine-needle aspiration for earlier diagnosis. Early referral and multidisciplinary approach may contribute to better management.

## Abbreviations

CT: Computed tomography
FNA: Fine-needle aspiration
MRI: Magnetic resonance imaging
